# The Comparative Treatment of Intraventricular Chemotherapy by Ommaya Reservoir vs. Lumbar Puncture in Patients With Leptomeningeal Carcinomatosis

**DOI:** 10.3389/fonc.2018.00509

**Published:** 2018-11-20

**Authors:** Mariano Montes de Oca Delgado, Bernardo Cacho Díaz, José Santos Zambrano, Vicente Guerrero Juárez, Manuel Salvador López Martínez, Elvira Castro Martínez, Javier Avendaño Méndez-Padilla, Sonia Mejía Pérez, Ignacio Reyes Moreno, Axayacatl Gutiérrez Aceves, Alberto González Aguilar

**Affiliations:** ^1^Emergency Department, National Institute of Neurology and Neurosurgery “Manuel Velasco Suárez” (INNN), Mexico City, Mexico; ^2^Neuroscience Unit, National Cancer Institute, Mexico City, Mexico; ^3^Neurosurgery Department, National Institute of Neurology and Neurosurgery “Manuel Velasco Suárez” (INNN), Mexico City, Mexico; ^4^Neurooncology Department, National Institute of Neurology and Neurosurgery “Manuel Velasco Suárez” (INNN), Mexico City, Mexico; ^5^Neurological Service, The American British Cowdray Medical Center, Mexico City, Mexico; ^6^Radioneurosurgery Department, National Institute of Neurology and Neurosurgery “Manuel Velasco Suárez” (INNN), Mexico City, Mexico

**Keywords:** leptomeningeal carcinomatosis, overall survival, chemotherapy, ommaya reservoir, lumbar puncture, karnofsky performance status, intraventricular

## Abstract

**Object:** Leptomeningeal Carcinomatosis (LCM) represents a state of systemic malignant disease with poor prognosis. The purpose of this study is to compare overall survival (OS) between intraventricular chemotherapy through Ommaya reservoir (OR) and chemotherapy through lumbar puncture (LP) in LCM.

**Patients and Methods:** Forty adult patients with LCM were included. All patients underwent lumbar puncture and Magnetic resonance imaging (MRI). Thirty patients received chemotherapy through LP and 10 undergone colocation of Ommaya reservoir for intraventricular chemotherapy.

**Results:** The most common symptom was headache (Present in 50%). The cranial nerves most affected were VI and VII. Leptomeningeal enhancement was the most frequent finding in MRI. The OS in the LP group was 4 months and Ommaya group was 9.2 months (*p* = 0.0006; CI:1.8-3), with statistical differences in favor to Intraventricular treatment. Proportional hazard regression showed that receiving chemotherapy through Ommaya reservoir was a protective factor (Hazard ratio = 0.258, Standard Error = 0.112, *p* = 0.002 and 95% CI 0.110-0.606). Using KPS as a factor did not affect the hazard ratio of Ommaya reservoir itself.

**Conclusions:** OS was significantly higher in patients with Ommaya reservoir in spite of Karnofsky Performance Status (KPS) previous to chemotherapy. Therefore, intraventricular chemotherapy should be preferred over lumbar puncture chemotherapy administration if there are resources available.

## Introduction

LCM is a rare complication of advanced cancer, which consists in infiltration of the meninges and Cerebrospinal fluid (CFS) space by malignant cells (1). Any cancer can metastasize to meninges but is mainly detected in association with breast cancer, lung cancer, melanoma, and in fewer occasions with other types of cancer (Gastrointestinal, prostate, lymphoma, leukemia, unknown primary cancer) (2). It has an incidence of ~5% of the patients with cancer but because of the asymptomatic patients or late-onset symptomatology, it may increase even to 20% as biopsies studies have demonstrated (3, 4). The median survival is around 4–6 weeks when untreated but it may improve as well as neurological status because of chemotherapy regimens (2, 4, 5). Karnofsky Performance Status (KPS) is the most reliable prognostic factor in patients with diagnosis of LCM (3, 4, 6). The gold standard remains the identification of malignant cells in CSF cytological study (7). The treatment goals are to improve the neurological status and to prolong survival. Different treatments are used (Radiotherapy and Neurosurgery) but the chemotherapy is essential in the management of LCM. Traditionally the method of election was the lumbar puncture (Intrathecal), but currently there are other options such as the Ommaya reservoir (Intraventricular) that might have better outcomes for patients (8). There is not a standard route of administration and both are recommended taking into consideration that chemotherapy needs good distribution and penetration; Intraventricular chemotherapy acts directly in CSF and probably it is superior to lumbar administration but there is not a trial that confirms this hypothesis completely. The present article is a retrospective study that compares the Intraventricular vs. the lumbar administration of chemotherapy in LCM.

## Patients and methods

We conducted a retrospective study collecting and analyzing data from patients diagnosed with LCM between 1980 and 2016 at National Institute of Neurology and Neurosurgery. We obtained clinical, imaging, histological, and treatment outcome data from electronic database such as gender, age, Karnofsky Performance Status (KPS), overall survival in months (OS, established with date of decease), symptomatology, primary tumor, localization of lesion by neuroimaging, treatment received, date of histological diagnosis, lumbar puncture (Glucose, proteins, cells, malignant cells), HIV status, and type of treatment (Intraventricular and Intrathecal). Diagnosis was established by presence of malignant cells in CSF and by neuroimaging findings in patients with histological diagnosis of cancer. Statistical analysis was performed using Stata/MP 14.1. In an effort to identify potential bias we stablished mean and *t*-test for scalar variables. Survival was established by Kaplan-Meier method taking on account impact of primary tumor and KPS. We used log-rank test to establish the statistical significance of difference in overall survival.

### Chemotherapy protocol

The chemotherapy regimen administered was Methotrexate 15 mg (MTX) monotherapy, and IT triple therapy (IT-triple; 15 mg MTX, 30 mg/m^2^ Cytarabine and 15 mg/m^2^ Hydrocortisone) or Cytarabine (Ara-C) alone 30 mg/m^2^. The regimen was administered up to twice a week, according to the condition of the patient, until negative cytology (Induction phase), followed by once weekly for 4 weeks (Consolidation phase) and the last maintenance phase was once a month until progression, maximal doses or death.

## Results

We identified 40 patients; ten patients had undergone installation of an intraventricular Ommaya reservoir (Between 2000 and 2014) and received chemotherapy for LCM while 30 patients received intrathecal chemotherapy through lumbar puncture. We obtained the following data: 26 patients were male (65%) and 14 were female (35%) with a ratio of 1.8:1. The median age was 52 years range of 18–76 (Table 1).

**Table 1:** Patients characteristics.

	**All patients**	**Ommaya**	**LP**	***p***
Gender	*n* = 40 %(n)	10 (25)	30 (75)	0.251
Male	26 (65)	5 (50)	21(70)
Female	14 (35)	5 (50)	9 (30)
Age in years, median (range)	52 (18-76)	50 (18-64)	54.5 (20-76)	0.168 (Xi2) 0.033 (Fisher)
KPS, median (range)	70 (40-100)	70 (50-100)	70 (40-100)	0.580
**PRIMARY TUMOR**				
•Breast	10 (25)	3 (30)	7 (23.33)
•Lung	7 (17.5)	0 (0)	7 (23.33)
•Leukemia	7(17.5)	2 (20)	5 (16.67)
•Melanoma	5 (12.5)	0 (0)	5 (16.67)
•Ovary	4 (10)	2 (20)	2 (6.67)
•Prostate	3 (7.5)	1 (10)	2 (6.67)
•Lymphoma	2 (5)	1 (10)	1 (3.33)
•Unknown	2 (5)	1 (10)	1(3.33)
**TREATMENT REGIMEN**				
•Ara-C	7 (17.5)	1 (10)	6 (20)	0.846
•Mtx	5 (12.5)	1(10)	4 (13.33)
•Mtx/Ara	28 (70)	8 (80)	20 (66.67)
Overall survival (OS in months)	0.4 – 10	3-10	0.4-7.1	0.0006

*KPS, Karnofsky Performance Status; LP, Lumbar puncture; Ara-C, Citarabine; Mtx: Methotrexate*.

The KPS range was 40–100, with median of 70. Neurological examination and clinical symptoms were as follows: Headache was the most common symptom, present in 20 patients (50%). The rest of signs and cranial nerves (CN) most affected as well as KPS previous treatment are resumed in Table 2.

**Table 2:** Clinical symptoms and cranial nerves affected.

**Clinical features**	**No. patients**	**Percentage**
Headache	20	50
Seizures (Tonic-clonic)	5	12.5
Nausea or vomit	8	20
Cognitive disorders	6	15
Altered state of consciousness	5	12.5
Motor	12	30
Sensitive	6	15
Cerebellum	5	12.5
Ataxia	5	12.5
Diplopia	8	20
Dysphagia	2	5
Dysarthria	3	7.5
Radicular pain	3	7.5
Cranial nerve affection	18	45
**Cranial nerves affection**	**No. patients**	**Percentage**
None	20	50
IX, X	3	7.5
VI	4	10
VI, III	3	7.5
VI, IX, X	2	5
VII	5	12.5
VIII	3	7.5
**KPS previous treatment**	**LP (%)**	**Ommaya (%)**
40	1 (3.3)	0
50	4 (13.3)	3 (30)
60	7 (23.3)	2 (20)
70	8 (26.6)	2 (20)
80	2 (6.6)	0
90	4 (13.3)	2 (20)
100	4 (13.3)	1 (10)
Total	30 (100)	10 (100)

**Figure 1:**
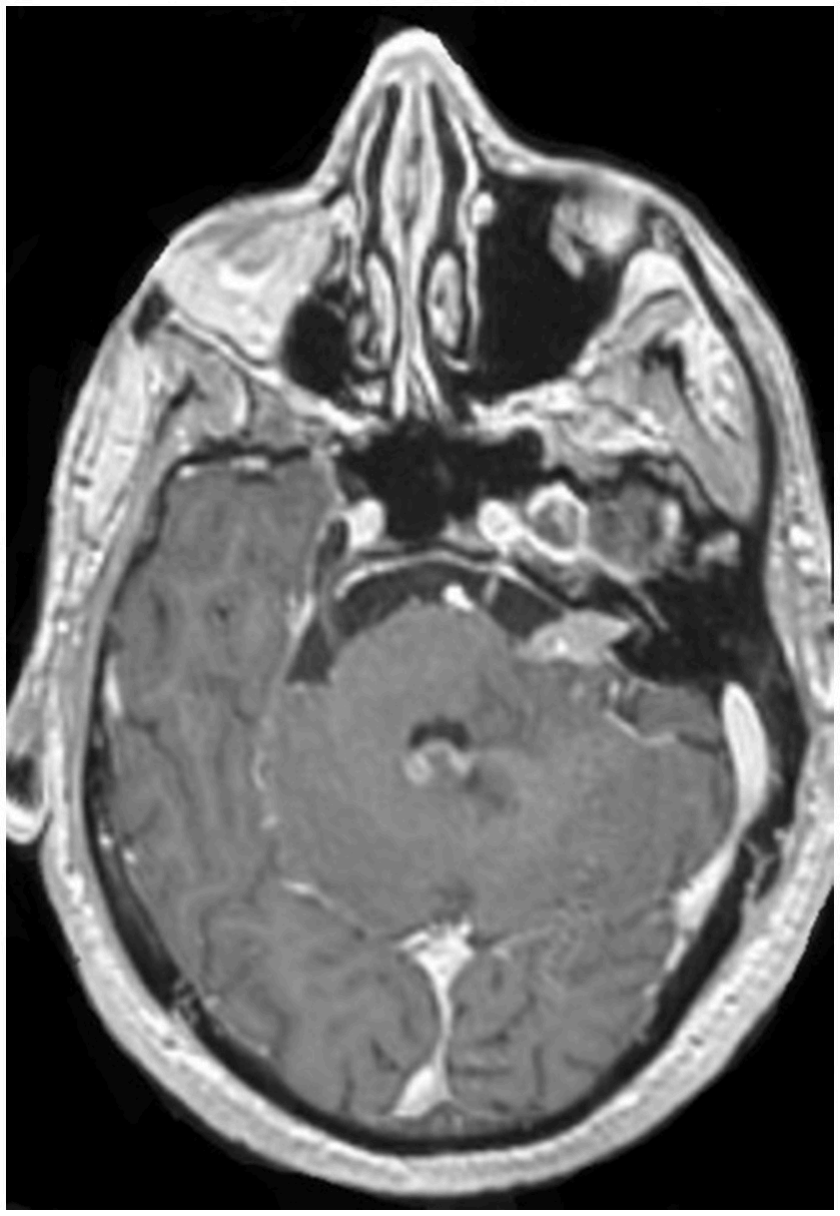
Axial MRI with nodular contrast enhancement in VII cranial nerve and enhancement in fourth ventricle, patient with diagnosis of lung cancer.

The most affected CN were VI and VII. Neuroimaging findings were: meningeal enhancement (especially in cerebellum 21/40 patients) and nodular lesions 15/40 patients (Figure 1), in the 30% of patients the Magnetic Resonance Imaging (MRI) was normal.

**Figure 2:**
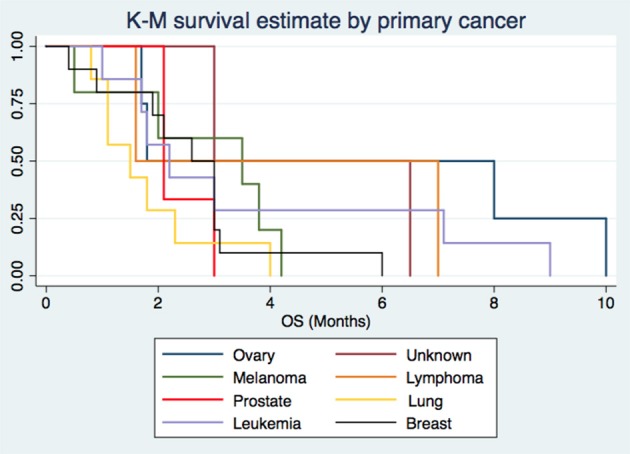
The origin of Leptomeningeal Carcinomatosis by primary cancer.

The most common site of primary tumor was breast with 10 patients (25%), followed by lung (7 patients, 17.5%) and Leukemia (7 patients, 17.5%), then melanoma with 5 patients (12.5%), ovary with 4 patients (10%), then prostate with 3 patients (7.5%) and at last but not least lymphoma and unknown with 2 patients (5%, respectively) (Figure 2).

The classic pattern of LCM is Hypoglycorrhachia, hyperproteinorrhachia, and malignant cells present. We analyzed the percentage of patients that presented this classic pattern and those who present different pattern. Referent to proteins in CSF, all patients presented values >45 (Range of 53–245), mean of 101.8. Glucose outcomes were divided in: Normal (8 patients, 20%), hypoglycorrhachia (30 patients, 75%) and hyperglycorrhachia (2 patients, 5%). Cellularity was present in all patients, with a range of 5–985, mean of 55.3. Classic pattern was present in 30 patients of the sample (75%).

**Figure 3:**
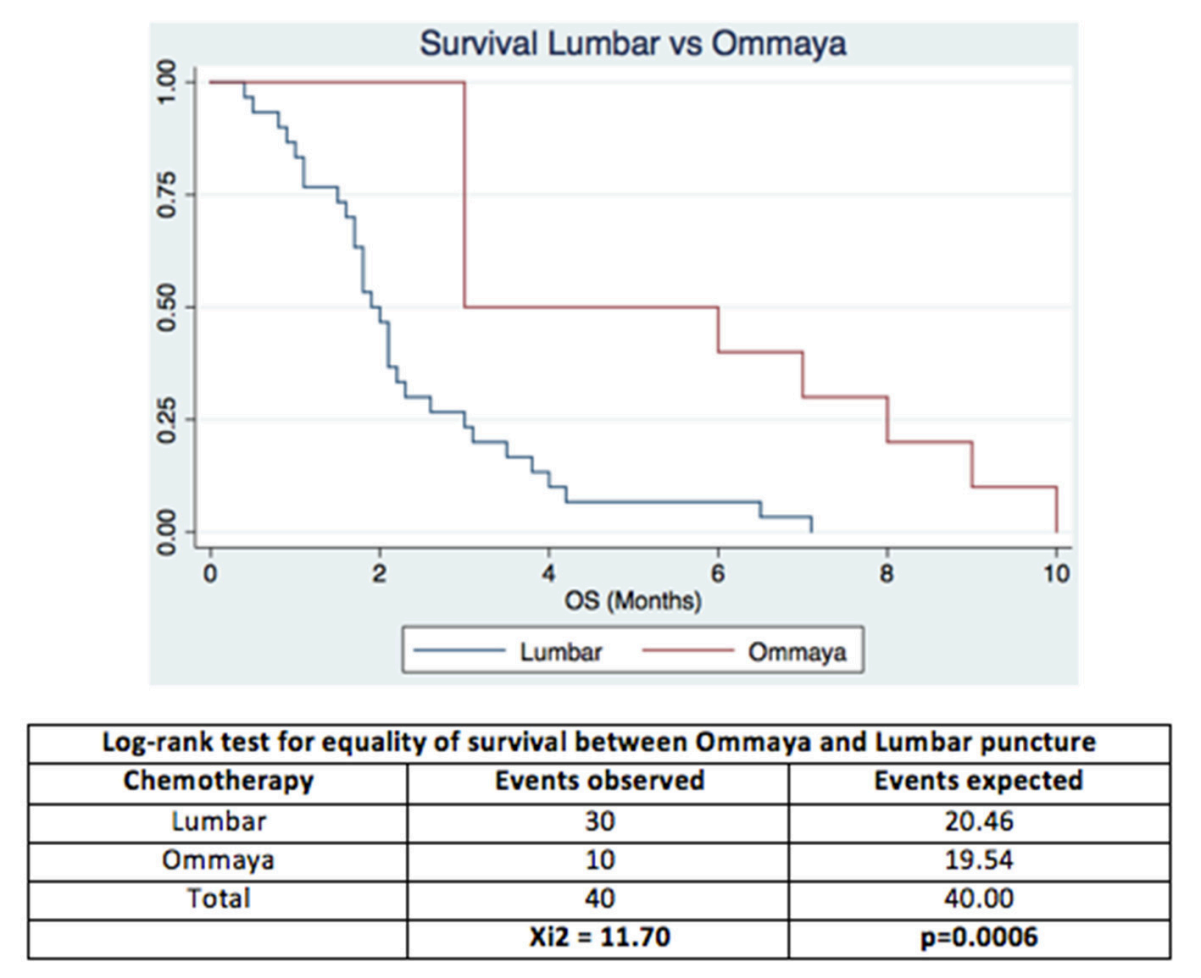
The Overall Survival by Lumbar chemotherapy vs. Intraventricular with Ommaya reservoir in patients with Leptomeningeal Carcinomatosis.

The Overall survival in the group with chemotherapy by LP was 4 months and Ommaya group was 9.2 months (*p* = 0.0006; CI:1.8-3), significantly higher in patients who received chemotherapy through Ommaya reservoir (see Figure 3).

Cox model for proportional-hazard regression showed that receiving chemotherapy through Ommaya reservoir was a protective factor (Hazard ratio = 0.258, Standard Error = 0.112, *p* = 0.002 and 95% CI 0.110-0.606). Using KPS as a factor did not affect the hazard ratio of Ommaya reservoir itself.

**Figure 4:**
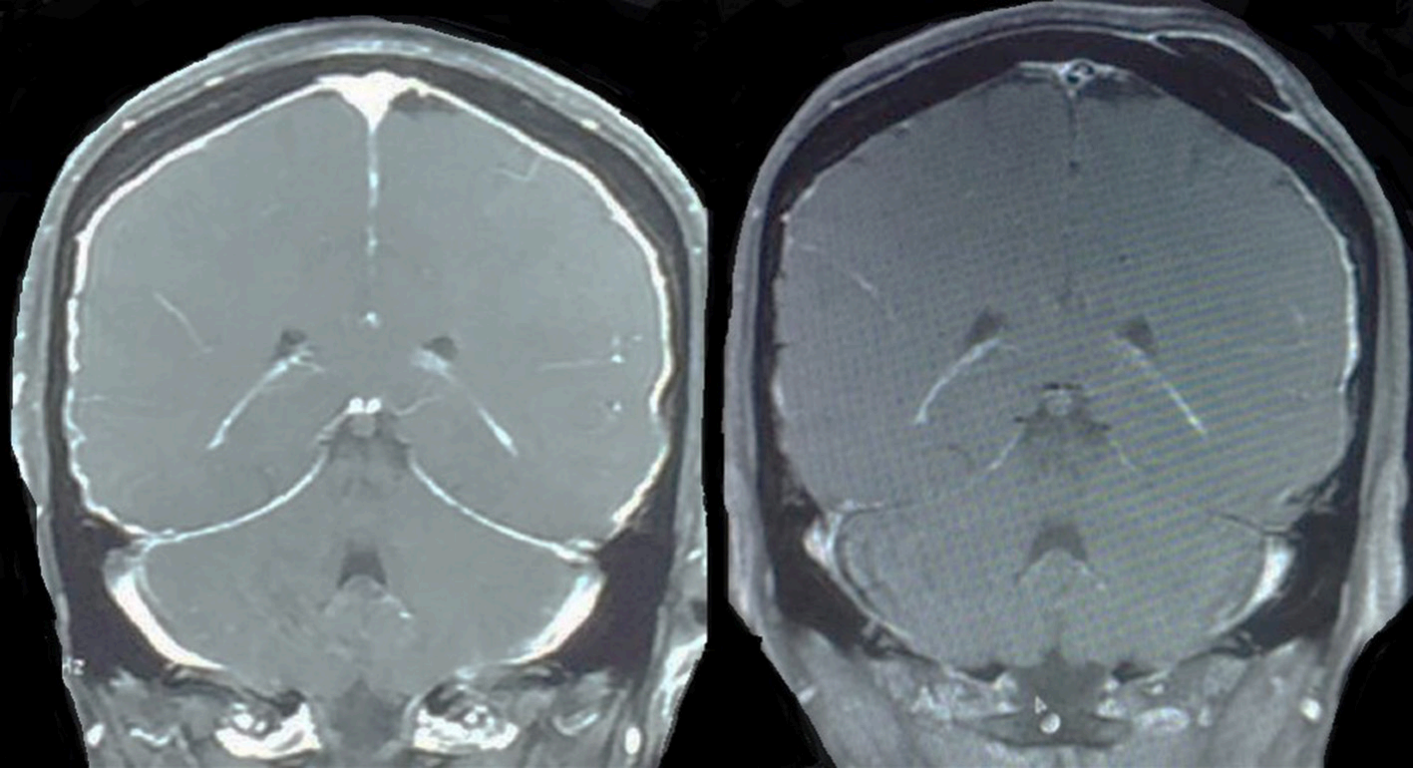
45 yr old female patient with diagnosis of breast cancer and leptomeningeal disease with complete radiological response after treatment with Mtx/Ara-C by Ommaya reservoir.

Overall survival by primary cancer had a median of 1.5 months for lung cancer, 2.1 months for prostate, 2.2 months for leukemia, 2.8 months for breast cancer, 3 months for melanoma, 4.3 months for lymphoma, 4.7 months for unknown primary, and 4.9 months for ovary cancer. Cox regression method showed breast and lung cancer as risk factors for poor prognosis with statistical significance (*p* = 0.069 and *p* = 0.012, respectively) (see Figure 4).

Regarding complications, two patients were reported with neuroinfectious disease with isolation and identification of *Klebsiella pneumoniae* in both cases. According to CTR (Common Toxicity Criteria) chemotherapy related complications noted were: Two patients with toxicity grade 2 in platelets count, two patients with toxicity grade 2 in WBC count, and one patient with toxicity grade 4 in platelet count.

We analyzed the prognostic factors (age, KPS, Chemotherapy and Intraventricular chemotherapy) without statistical differences (see Table 3).

**Table 3:** Favorable prognostic factor in Leptomeningeal carcinomatosis by Cox regression method.

**Cox regression**						
**No. subjects** = **40**			**No. observations** = **40**		
**No. failures** = **40**
**Variable**	**Hazard ratio**	**Standard error**	**z**	***p***	**95% Conf. Interval**	
KPS	1.013	0.121	1.10	0.270	0.989	1.037
Ommaya	0.259	0.112	−3.11	0.002	0.111	0.607
Triple chemotherapy	1.230	0.134	1.32	0.906	0.896	1.506

## Discussion

LCM is a rare complication of advanced cancer, which consists in infiltration of the meninges and CFS space by malignant cells and with the presence of new treatments that increase survival it is likely that its frequency increases. We have reported and analyzed the outcomes obtained with chemotherapy through lumbar puncture vs. Ommaya reservoir in patients with diagnosis of LCM. There are numerous topics to underline. Some of our results were consistent with those reported on previous clinical trials.

As we mentioned before, headache was the most common symptom, present in 20 patients (50%), which is concordant with studies that described clinical features in LCM (9, 10). Cranial nerves are usually affected in patients with LCM, our patients presented mainly affection of VI and VII nerves alone or in combination with other cranial nerves (35% in total). Some reviews have noted that indeed, VI is the cranial nerve most affected (8, 11).

Previous studies showed abnormalities in CSF in more than 90% of the cases (11–13). It is necessary to have in mind that the most frequent pattern profile in lumbar puncture in patients with LCM began to be well identified since the 50's, nowadays CSF analysis has great importance in the approach of LCM (14–16). All of our patients presented abnormalities in CSF and 75% of them presented the classic pattern that we had discussed.

LCM involves the entire axis of CNS, therefore MRI takes great relevance. Contrast enhancement is necessary when obtaining a neuroaxis image. The principal site of lesion in our study was by far cerebellum, but it is clear that sites affected were variable and did not follow a pattern. However, bulky lesions are not always observable and diffuse pattern can be present as well as multiple lesions (17–19).

There are only three drugs available to administer intra-CSF: Methotrexate (MTX), Cytarabine (Ara-C) and less often Thiotepa. Effectiveness of these drugs is demonstrated in LCM, nevertheless is limited in some solid tumors associated to LCM (Melanoma and lung cancer). There was not significant difference between the three distinct types of chemotherapy employed in our study (Methotrexate, Liposomal Cytarabine and MTX/Ara-C). Until now, effectivity of these drugs remains similar (3, 20). In addition, combination vs. single agent therapy neither has shown overwhelming superiority so it remains controversial, but it can be associated to less tolerance (14, 21). OS was significantly higher in the Ommaya reservoir group. The patient with the highest OS was a young woman with low KPS, nevertheless the log-rank showed no significance to this point. Concerning to Intraventricular chemotherapy administration, there is adequate drug distribution within the leptomeningeal space. Even when CSF flow is unimpeded, the normal CSF circulation carries fluid preferentially to the ventricles (22, 23). As a result, the delivery of drug administered into the lumbar Intrathecal space is unlikely to achieve clinically relevant drug concentrations within the cerebral ventricles, where malignant cells are known to reside (24–27).This may explain the observation that there is better response in patients who receive Intraventricular chemotherapy, in contrast to Intrathecal chemotherapy.

On the other hand, a recent clinical phase II trial (28), demonstrates that disturbances in the CSF flow makes chemotherapy ineffective as it may hinder the drug distribution and increase intracranial pressure. Same authors have pointed that ventroculolumbar chemotherapy showed improvement of increased intracranial pressure, altered mental status and cauda echina symptoms. It must be noted that this trial only includes Methotrexate in evaluation, however is a reliable study about perfusion rate, adverse effects and toxicity.

About primary tumors, results were similar to the rest of literature. Confirming that breast cancer, lung cancer and melanoma are the solid tumors with major association to LCM (16).

OS was affected by primary cancer as other studies have demonstrated (3, 29, 30).

Data regarding complications related to placement of Ommaya reservoir was limited to those noted on database.

## Conclusions

In our study, OS was determined by the factors previously mentioned, which is consistent with reports on similar trials. LCM represents an advanced stage of cancer and therefore it is a pathology of poor prognosis. Analysis of CSF and MRI to identify sites of lesions are fundamental to achieve diagnosis and to establish management. Recent research indicates that the future in the treatment of LCM is in the study of molecular targeted therapies such as epidermal growth factor receptor (EGFR) and anaplastic lymphoma kinase (ALK) (31).

Another diagnostic studies like rare cell capture technology should be taken into consideration to the approach of LCM on the future. As well as the detection of CSF malignant cells through CellSearch (32, 33).

Chemotherapy is the cornerstone of the treatment and Intraventricular administration through Ommaya reservoir or other dispositive, have shown similar outcomes and also have demonstrated to be the best option when available.

We should mention that our results stablish intraventricular chemotherapy as a better option of treatment in this group of patients, nevertheless, due to the retrospective design and extended time of study, result should be taken cautiously. Further studies must include bigger sample size with data about complications related to the procedures such as increased intracranial pressure and ventriculitis.

A prospective randomized study would be ideal to set conclusions but we consider it particularly difficult to select a homogeneous sample in patients with leptomeningeal disease.

Is important always having in mind that the objective of chemotherapy is to improve neurological status and quality of life more than prolonging survival. Next trials should be focused on improving diagnostic and therapeutic options that may reduce costs, avoid delayed processing, exempt patients from invasive procedures and allow a more precise diagnosis and prognosis.

## Ethics statement

Ethical review process was not required due to the retrospective design of study and anonymized data of patients included, in accordance with the local legislation and institutional requirements.

## Author contributions

MM and AGo: design of study, data collection, manuscript writing, analysis of results, discussion. BC, JS, VG, ML, EC, JA, SM, IR, and AGu: manuscript writing, analysis of results.

### Conflict of interest statement

The authors declare that the research was conducted in the absence of any commercial or financial relationships that could be construed as a potential conflict of interest.
